# An Improved POD Model for Fast Semi-Quantitative Analysis of Carbendazim in Fruit by Surface Enhanced Raman Spectroscopy

**DOI:** 10.3390/molecules27134230

**Published:** 2022-06-30

**Authors:** Qiaoling Yang, Hong Lin, Jinge Ma, Niannian Chen, Chaomin Zhao, Dehua Guo, Bing Niu, Zhihui Zhao, Xiaojun Deng, Qin Chen

**Affiliations:** 1 School of Environmental and Chemical Engineering, Shanghai University, Shanghai 200444, China; yangqiaoling@shu.edu.cn; 2 School of Life Sciences, Shanghai University, Shanghai 200444, China; bingniu@shu.edu.cn; 3 Department of Endocrine and Metabolic Diseases, Shanghai Institute of Endocrine and Metabolic Diseases, Ruijin Hospital, Shanghai Jiao Tong University School of Medicine, Shanghai 200025, China; lh12263@rjh.com.cn; 4 Tech Ctr Anim Plant & Food Inspect & Quarantine, Shanghai Customs, Shanghai, 200135, China; 15678092339@163.com (J.M.); n_smily@163.com (N.C.); chaominzhao@126.com (C.Z.); guodehua@customs.gov.cn (D.G.); 5 Shanghai Oceanhood Instrument Equipment Co., Ltd., Shanghai 201608, China; zhihui.zhao@oceanhood.com; 6 School of Kinesiology, Shanghai University of Sport, Shanghai 200438, China

**Keywords:** surface-enhanced Raman spectroscopy, carbendazim, probability of detection model, semi-quantitative analysis, rapid detection method evaluation

## Abstract

The current detection method of carbendazim suffers from the disadvantages of complicated preprocessing and long cycle time. In order to solve the problem of rapid quantitative screening of finite contaminants, this article proposed a qualitative method based on characteristic peaks and a semi-quantitative method based on threshold to detect carbendazim in apple, and finally the method is evaluated by a validation system based on binary output. The results showed that the detection limit for carbendazim was 0.5 mg/kg, and the detection probability was 100% when the concentration was no less than 1 mg/kg. The semi-quantitative analysis method had a false positive rate of 0% and 5% at 0.5 mg/kg and 2.5 mg/kg, respectively. The results of method evaluation showed that when the added concentration was greater than 2.5 mg/kg, the qualitative detection method was consistent with the reference method. When the concentration was no less than 5 mg/kg, the semi-quantitative method is consistent between different labs. The semi-quantitative method proposed in this study can achieve the screening of finite contaminants in blind samples and simplify the test validation process through the detection probability model, which can meet the needs of rapid on-site detection and has a good application prospect.

## 1. Introduction

Carbendazim (CBZ) is a broad-spectrum fungicide that is effective against diseases caused by fungi (e.g., Demodex, Polychaeta) in various crops. However, its residues are toxic to mammals and can cause liver disease and chromosomal aberrations [1,2]. Prashantkumar et al. found that exposure to CBZ in male goats caused testicular damage and impaired liver, kidney and blood function [3]. Meanwhile, the study found that CBZ can change the antioxidant defense system [4]. Hence, in the field of food safety, CBZ is restricted in different countries and foods. China stipulates that the maximum residue limit (MRL; The maximum legal allowable residue concentration of pesticides in an agricultural product, food and feed.) of CBZ in apple is <5 mg/kg [5], and the EU stipulates that the MRL is <2 mg/kg [6]. The existing detection methods of CBZ include high performance liquid chromatography (HPLC) [7], liquid chromatography-tandem mass spectrometry (LC-MS/MS) [8], electrochemistry [9], immunosensor [10] and so on. Lee et al. used QuEChERS and LC-MS/MS to simultaneously detect the concentrations of thiophanate-methyl and CBZ in pears. The results showed that the detection limit of CBZ was as low as 0.0012 mg/kg [11]. Liu et al. applied HPLC with fluorescence detection to determinate CBZ and thiabendazole in apple juice. The limit of detection was 0.8 µg /kg for CBZ [12]. Although these detection techniques are highly sensitive and reliable, their preprocessing is complex, time-consuming and costly. Furthermore, these detection techniques cannot perform rapid detection for a large number of samples. Nowadays, rapid detection techniques including near-infrared spectroscopy, Raman spectroscopy, hyperspectral, etc. have been widely applied in the field of food quality and safety monitoring. Among them, Surface-enhanced Raman spectroscopy (SERS) [13,14] developed rapidly due to its outstanding sensitivity, specificity, real-time response and “fingerprint” identification characteristics, and is widely performed in food safety assessment [15], especially the qualitative analysis of finite contaminants food [16,17], including antibiotics [18], metal ions [19], microorganisms [20], pesticides [21], etc. For example, Chen et al. detected CBZ in oolong tea by SERS, and used the partial least squares method to quantitatively analyze the content of CBZ. The results showed a good linear relationship between the spiked and predicted carbendazim in methanol–water solution (R = 0.972; slope = 0.975; RMSEP = 0.819 mg/L) [22].

Quantitative analysis of limited finite contaminants has been widely applied in the field of food finite contaminants analysis [23]. The quantitative analysis of Raman spectroscopy is based on a linear proportional relationship between the Raman characteristic peak intensity and the concentration of the analyte. Although SERS technology has high sensitivity and advantages in the field of trace detection, the Raman peak intensity detected is affected by some factors such as the randomness of surface-enhanced particle aggregation, matrix, sample transparency and so on [24]. Hence, Raman peak intensities collected by SERS cannot form a clear linear proportional relationship with the concentration. To resolve this problem, existing standard curve-based quantification methods need to be improved according to the requirements of rapid testing. Recent studies suggested that the Raman peak intensity detected by SERS conforms to a Gaussian distribution [25], which inspired us to use Raman characteristic peak intensity of CBZ for semi-quantitative modeling to achieve rapid screening of limited finite contaminants, thereby avoiding complex quantitative analysis of SERS and improving detection efficiency.

As one of the rapid detection methods for food, SERS technology requires implemental technical specifications to validate its applicability in the screening of food finite contaminants. Both qualitative and semi-quantitative SERS assays are binary outputs, which can be evaluated by a validation system based on binary outputs. At present, there are many methods that can effectively evaluate the accuracy of binary results, such as Wilrich based on ISO 5725 [26], Fleiss’s Kappa statistics [27], probability of detection (POD) model [28], Wieringen [29] and so on. POD is a model that reflects the change of detection probability with concentration [30] which can characterize metrics such as sensitivity, specificity, false positive and false negative rates. The POD model can plot qualitative data as a centralized function, and coordinate with the statistical parameters of quantitative method validation simultaneously, providing a unified statistical method for all method validation, solving the statistical problem of the unpaired test part. The evaluation process is concise, efficient, easy to understand, and implemented by non-statisticians, which has been successfully applied to the validation of chemical and microbiological methods [31,32] and to assess the reliability of non-destructive testing [33]. The evaluation method of SERS based on POD model not only satisfied the requirements of regulations, but also can make up for the poor quantitative ability of SERS and the lack of applicable evaluation methods.

Here, the intensity of Raman signature peak at the MRL of CBZ is used as the threshold, and the result is a quickly and effective method of determining whether the added concentration exceeds the MRL. Based on POD model, a standard SERS assay validation system was established to promote the commercial application of SERS in the field of rapid food detection.

## 2. Results

### 2.1. Establishment of a Rapid Detection Method for CBZ in Apple

#### 2.1.1. Selection of Raman Characteristic Peaks

**Figure 1:**
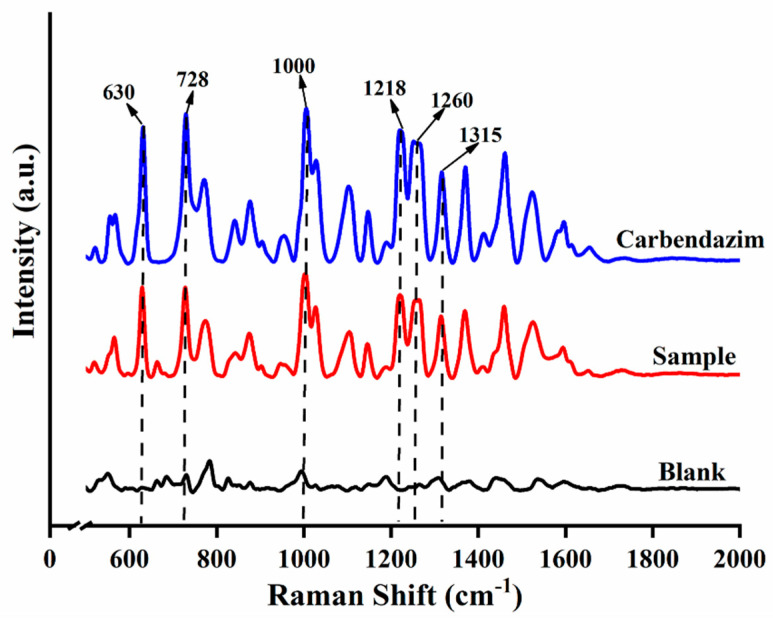
Raman spectra of different samples.

The SERS results of CBZ standard, spiked sample (5 mg/kg) and blank sample are shown in Figure 1. Six Raman shift peaks (630 cm^−1^, 728 cm^−1^, 1000 cm^−1^, 1218 cm^−1^, 1260 cm^−1^ and 1315 cm^−1^) were observed for CBZ standard and spiked sample compared to the blank sample. The six Raman shift peaks can be used as the qualitative characteristic peaks of CBZ in apple due to the clear peak shapes and distinct intensities. According to the SERS spectrum of CBZ and referring to the relevant literature [34,35,36]., the assignment of Raman shift and Vibrational Description were calculated and shown in Table 1.

#### 2.1.2. Establishment of POD Model

Based on the Raman characteristic peaks of CBZ, the detection of samples with different spiked concentrations was counted (Table 2). The results showed that the higher the added concentration, the greater the POD of CBZ in the spiked samples. When the added concentration was no less than 0.5 mg/kg, the POD range was (0.975, 1), which meets the requirement of the POD (>0.95). Hence, the LOD of this method was determined to be 0.5 mg/kg.

**Figure 2:**
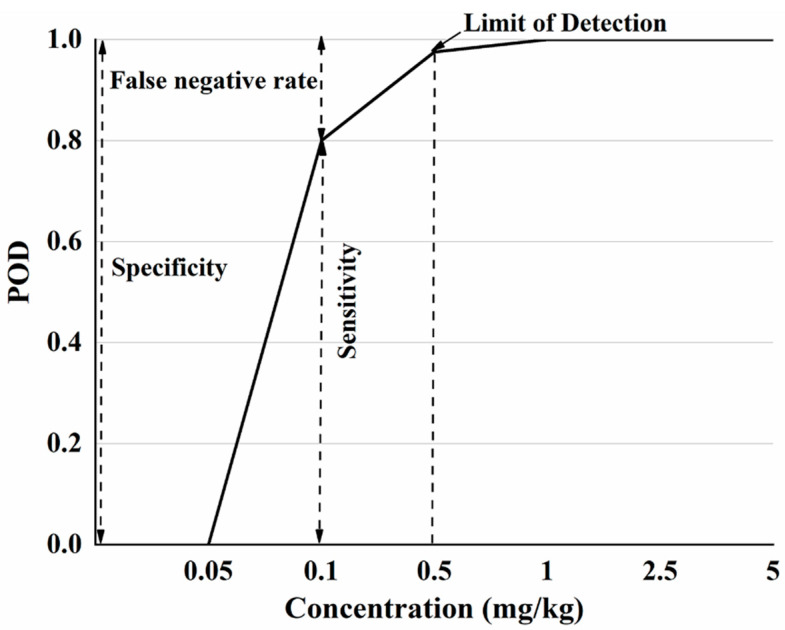
The POD model diagram of SERS detection method for CBZ in apple.

Based on the Table 2, a POD model was established with the additive concentration of CBZ in apple as the x-axis and the POD under different additive concentrations as the y-axis (Figure 2). According to ‘Technology specification for the evaluation of food rapid detection products’ (DB36/T 1334-2020) [37], when the sensitivity of the rapid detection method is greater than 95%, the additive concentration is LOD. When the added concentration is 0 mg/kg, this corresponds to a false positive rate for POD, where the sum of specificity and false positive rate is 100%. When added at concentration other than 0 mg/kg, sensitivity and POD were equal and the sum of the false negative rate and sensitivity is 100%.

Figure 2 shows that when the sample does not contain CBZ, the POD is 0, indicating that the false positive rate of this method is 0. Since the sum of the false positive rate and specificity is 100%, so the specificity is 100%. When the additive concentration is not more than 0.05 mg/kg, the detection probability is 0, the sensitivity of the method at this concentration is 0, and the false negative rate is 100%. When the additive CBZ concentration is in the range of 0.05 mg/kg–1 mg/kg, the POD is different under different additive concentrations, the sensitivity and false negative rate are different, of which 0.5 mg/kg (sensitivity > 95%) is the LOD. When the added concentration is greater than 1 mg/kg, the POD is 100%. Based on the POD model plots, the specificity, sensitivity, false positive and false negative rates of SERS detection of CBZ in apples at different spiked concentrations can be visually analyzed.

#### 2.1.3. Consistency Evaluation of Qualitative Methods Based on POD Model

(1) Consistency evaluation between qualitative method and reference method

At different additive concentrations, the qualitative POD of SERS method and HPLC [38] (GB/T 23380-2009) is shown in Table 3. It can be seen from Table 3 that when the concentration of CBZ is less than 0.01 mg/kg, the POD of the two methods is 0, and the dPOD is 0. The POD of the SERS method was lower than that of the HPLC method when the additive concentration was in the range of (0.01,2.5) mg/kg, and the POD of the HPLC was 100%. When the additive concentration was not lower than 2.5 mg/kg, the POD of both the SERS method and the reference method was 100%, and the dPOD is 0. This suggests that the SERS qualitative assay method has good reproducibility and is the same as the reference method.

**Figure 3:**
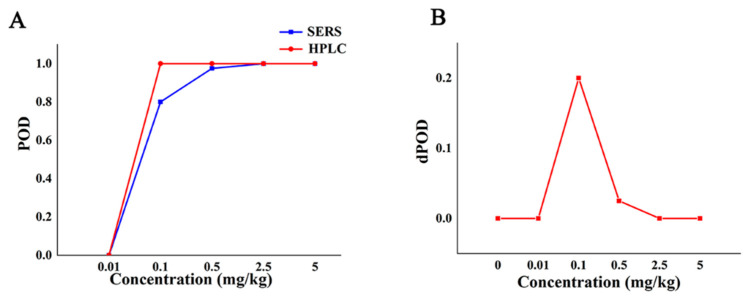
The detection situation of CBZ in apple by Raman qualitative method and reference method. Note: (**A**,**B**) are the POD curve and the dPOD curve of the Raman qualitative detection method and the reference method, respectively.

The POD curve and dPOD curve of Raman qualitative method and reference method were obtained (Figure 3) according to the consistency evaluation table of Raman qualitative method and reference method for CBZ in apple (Table 3). Based on the analysis of the POD (Figure 2), the false positive rate of SERS and HPLC in Figure 3A is 0, and the specificity is 100%. When the additive concentration was between 0.01 mg/kg and 2.5 mg/kg, the false negative rate of SERS was higher than that of HPLC. When the additive concentration was 5.0 mg/kg, the POD of SERS and HPLC are both 100%, the sensitivities are both 100% (Figure 3A). It can be seen from Figure 3B that when the added concentration is no less than 2.5 mg/kg, the dPOD of the two detection methods is 0, indicating that the SERS qualitative detection method and the reference method have the same detection results. This suggests that the SERS qualitative assay can meet the needs of limited CBZ detection at the MRL level of CBZ (5 mg/kg), while improving detection efficiency.

(2) Consistency evaluation of qualitative method among different labs

Table 4 shows the qualitative POD of SERS detection method between two different labs with different additive concentrations. The environments of lab I and lab II are different, and the same Raman instrument was always used during the experiment and the acquisition parameters were the same. The results showed that the POD of the Raman qualitative method in the two labs was 0 when the additive concentration was not more than 0.05 mg/kg. When the added concentration was in the range of 0.05 mg/kg to 2.5 mg/kg, the POD between the two labs was different, and the POD of lab II was higher than that of lab I. When the additive concentration was no less than 2.5 mg/kg, the POD between the two labs was 100%, and the dPOD was 0, which shows that the Raman qualitative method has the same detection results among different labs and has good repeatability.

**Figure 4:**
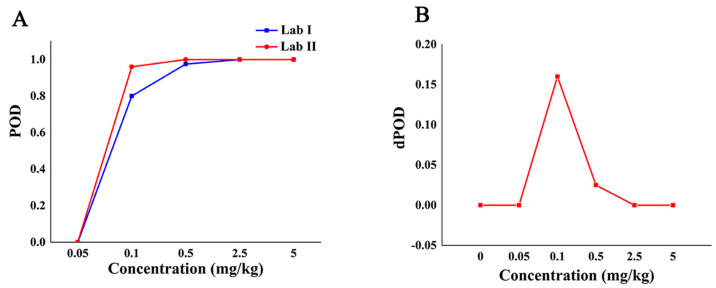
The detection situation of CBZ in apple by Raman qualitative method in different labs. Note: (**A**,**B**) are the POD curve and the dPOD curve of the Raman qualitative detection method between different labs, respectively.

The POD curve and the dPOD curve between different labs were obtained based on the POD of the qualitative methods between the different laboratories (Figure 4). Compared with the analysis results in Figure 3A, it can be seen from Figure 4A that the false positive rate of the method between different labs is 0, and the specificity is 100%. When the concentration is from 0.05 mg/kg to 2.5 mg/kg, the method had higher false negatives in lab I and higher sensitivity in lab II. When the concentration is no less than 2.5 mg/kg, the dPOD is 0 (Figure 4B), indicates that the detection results of this method between different labs are consistent. In other words, at the MRL (5.0 mg/kg) of CBZ, the Raman qualitative detection method can meet the requirements of limited detection and the results are not affected by the environment.

### 2.2. Establishment of a Semi-Quantitative Analysis Method for Carbendazim in Apple

The SERS rapid detection method allows accurate and qualitative analysis based on the characteristic peaks of CBZ. However, as CBZ is a finite contaminant in food, screening for finite contaminant concentrations is necessary for practical applications. In order to further judge whether the concentration of SERS qualitative detection reaches the MRL of CBZ, a semi-quantitative analysis method of CBZ based on Raman intensity threshold was developed. The method can determine whether the concentration of CBZ in the sample exceeds the MRL according to the characteristic peak intensity, which can avoid complex quantitative analysis and meet the needs of actual finite contaminant detection.

#### 2.2.1. Establishment and Screening of Semi-Quantitative Models

**Figure 5:**
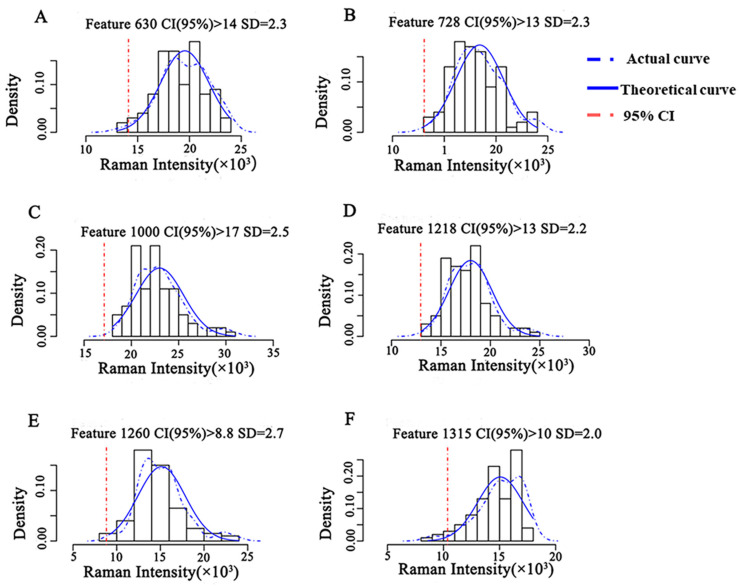
Semi-quantitative model of CBZ in apple at the MRL (5 mg/kg). Note: (**A**–**F**) represent the semi-quantitative models established by different Raman shifts, respectively. (**A**) 630 cm^−1^; (**B**) 728 cm^−1^; (**C**) 1000 cm^−1^; (**D**) 1218 cm^−1^; (**E**) 1260 cm^−1^; (**F**) 1315 cm^−1^. SD: Standard deviation.

The intensity distribution of the characteristic peaks of CBZ was obtained at the MRL level (5 mg/kg) based on the Raman spectral information of the semi-quantitative model training set (Figure 5). The actual intensity distribution was fitted with the theoretical Gaussian distribution, and the theoretical threshold for semi-quantitative analysis was obtained. The results showed that the actual distribution curve of Raman intensity at 630 cm^−1^ fit well with the theoretical Gaussian curve, and the fitting degree of the remaining five characteristic peaks was poor. When the CI was higher than 95%, the semi-quantitative model had a Raman intensity threshold of 1.4 × 10^4^ at 630 cm^−1^, indicating that the concentration of CBZ in apple was no less than 5 mg/kg when the intensity of characteristic peak at 630 cm^−1^ of CBZ in apple was greater than 1.4 × 10^4^.

**Figure 6:**
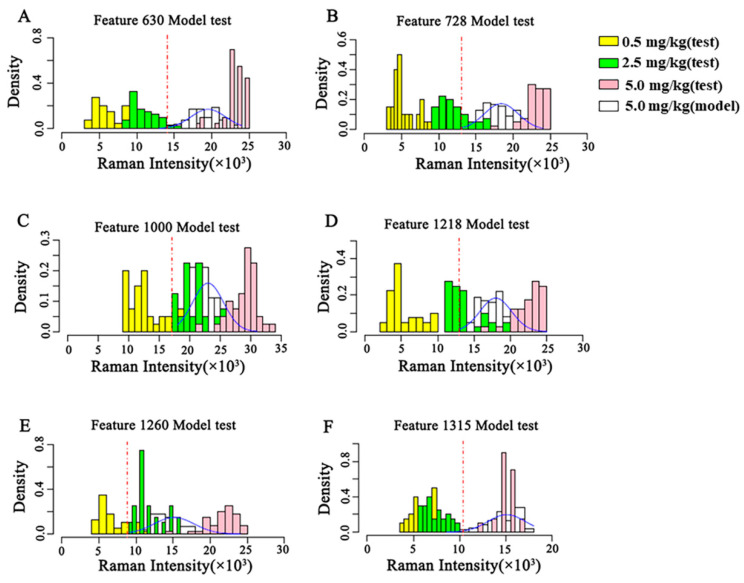
Validation of the semi-quantitative model on the MRL (5 mg/kg) of CBZ in apple. (**A**–**F**) represent the validation of semi-quantitative models established by different Raman shifts, respectively. (**A**) 630 cm^−1^; (**B**) 728 cm^−1^; (**C**) 1000 cm^−1^; (**D**) 1218 cm^−1^; (**E**) 1260 cm^−1^; (**F**) 1315 cm^−1^. SD: Standard deviation.

The concentration discrimination results of the three test sets of the semi-quantitative model are shown in Figure 6 based on these semi-quantitative models of CBZ in apple at the MRL (5 mg/kg). As shown in Figure 6, the discriminant results of the semi-quantitative models established by different characteristic peaks are different. The semi-quantitative model established at 630 cm^−1^ can well distinguish 0.5 mg/kg, 2.5 mg/kg and 5 mg/kg, and the concentration distribution results discriminated by the model are consistent with the reality. The test sets of the semi-quantitative models established at the remaining characteristic peaks have different degrees of overlap, and the results of model discrimination do not match the actual ones.

According to the Raman intensity thresholds of different semi-quantitative models (Figure 5) and the validation results of different semi-quantitative models (Figure 6), the POD that the concentration of CBZ in the three test sets exceeds the MRL and the scores of different semi-quantitative models are shown in Table 5. According to the calculation Formula (1) of the score, POD3 and score raised along with decrease of POD1 and POD2. Therefore, the higher the score of the model, the lower the false positive rate of the model, the higher the sensitivity, the better the model. The semi-quantitative model scores at 630 cm^−1^ and 1315 cm^−1^ are 97 and 100, respectively. However, the semi-quantitative model at 1315 cm^−1^ cannot distinguish the spiked samples at 0.5 mg/kg and 2.5 mg/kg (Figure 6). The semi-quantitative model at 630 cm^−1^ was well fitted (Figure 5) and the concentration distribution is consistent with reality, so the semi-quantitative model at 630 cm^−1^ is chosen as the optimal model for the semi-quantitative analysis of CBZ in apple. The model has a false positive rate of 0 at 0.5 mg/kg, a false positive rate of 5% at 2.5 mg/kg, and a POD of 100% at 5 mg/kg.

#### 2.2.2. Consistency Evaluation of Semi-Quantitative Methods among Different Labs

Table 6 shows the detection probability (whether the concentration of CBZ exceeds the MRL) of the semi-quantitative method in different labs under different additive concentrations. As shown in Table 6, when the added concentration is less than 5 mg/kg, the POD of the semi-quantitative method is different among different labs, and the POD of lab II is higher than that of lab I. When the additive concentration is 5 mg/kg, the POD of the semi-quantitative methods among different labs is 100%, and the dPOD was 0. Although the semi-quantitative model has higher false positives in lab II, it can accurately determine whether the concentration of CBZ exceeds the MRL between the two labs, and the repeatability is good.

**Figure 7:**
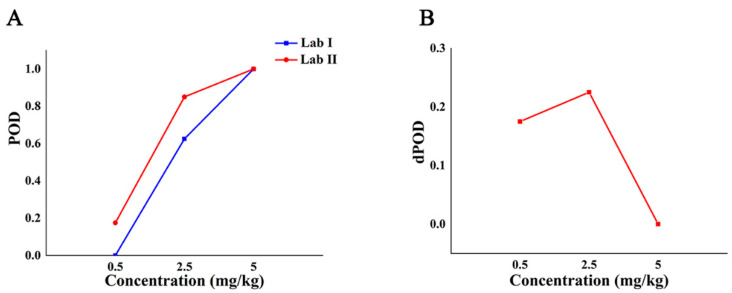
The detection situation of CBZ in apple by semi-quantitative method in different labs. Note: (**A**,**B**) are the POD curve and dPOD curve of semi-quantitative detection method between different labs, respectively.

According to the POD of the semi-quantitative method between labs, the POD curve and the dPOD curve between different labs were obtained (Figure 7). It can be seen from Figure 7A that when the added concentration is less than 5 mg/kg, the POD of the semi-quantitative method in different labs is different which suggested that the sensitivity of lab II is higher than that of lab I if sensitivity is different. The POD of both labs is 100% at 5 mg/kg. As shown in Figure 7B, when the concentration of CBZ in the sample is no less than 5 mg/kg, dPOD = 0, which indicates that the semi-quantitative method has the same detection situation among different labs. Therefore, the semi-quantitative method can effectively distinguish whether the additive concentration of the blind sample exceeds the MRL according to the intensity of the characteristic peak at 630 cm^−1^, and the results are not affected by the environment.

## 3. Discussion

CBZ is widely used in agriculture and is a common pesticide residue that threatens human and animal health. At present, some classical detection methods such as HPLC [7] can provide accurate qualitative and quantitative analysis of CBZ, but these traditional methods all involve complex pretreatment process, long detection cycles and complicated instrumentation. In order to meet the needs of rapid screening of finite contaminants in practical applications, it is necessary to develop a rapid detection method with a short detection cycle and simple operation.

SERS has been widely performed in the qualitative, quantitative and semi-quantitative analysis of food finite contaminants due to its specificity, sensitivity, non-destructive sample, and no interference from aqueous solutions [39]. The basis of SERS qualitative analysis is based on the Raman characteristic peaks of the target, selecting the characteristic shifts of the spiked sample and the target but not in the blank matrix as the characteristic peak of the target. In this study, the peak intensities at 630 cm^−1^, 728 cm^−1^, 1000 cm^−1^, 1218 cm^−1^, 1260 cm^−1^ and 1315 cm^−1^ in the CBZ standard and spiked samples were obvious. Therefore, these six Raman shifts were selected as the Raman characteristic peaks of CBZ in apple. The characteristic peaks of CBZ obtained here are the same as those of existing research [34]. For example, the peak at 630 cm^−1^ is related to the C–C–C in-plane bending and the peak at 728 cm^−1^ is attributed to the out-of-plane bending of the C-H bond in the benzene ring. The LOD of CBZ was 0.5 mg/kg, which was lower than the MRL of CBZ in apple (5 mg/kg). At the same time, it is four times lower than the LOD (2 mg/kg) of previous research methods [40].

Quantitative analysis of SERS is of great significance in the detection of finite contaminants in food. However, the Raman intensity stability of SERS is easily affected by enhanced matrix activity and environment, which makes quantitative analysis difficult. To overcome these issues, existing researches mainly focused on developing curing techniques to improve the stability and reproducibility of reinforced matrices. For example, Sivashanmugan et al. [41]. developed novel Au nanodot arrays on graphene substrates for highly active enhanced Raman scattering. By using Rhodamine 6G (R6G) as a molecular probe, the LOD was as low as 10^−12^ M and the Raman enhancement factor was as high as 10^8^. Wu et al. developed a simple and effective SERS tape based on biconical gold nanoparticles (BP-AuNPs) for monitoring methyl parathion residues on the surfaces of vegetables and fruits [21]. In real world applications, the screening of finite contaminants in food mainly depends on the MRL. If the added concentration of the sample is higher than the MRL, it is judged as a non-conforming product, otherwise it is judged as a qualified product. In order to fulfill the detection requirements of finite contaminants, we developed a threshold-based semi-quantitative analysis method for finite contaminants, which reduces the difficulty and cost of developing new materials and improves the detection efficiency. The model established at 630 cm^−1^ was selected as the basis for semi-quantitative analysis of CBZ in apple after screening and verification. When the Raman intensity at 630 cm^−1^ was greater than 1.4 × 10^4^, the concentration of CBZ in the sample was higher than the MRL (5 mg/kg). When the additive concentration was 5 mg/kg, the POD of this semi-quantitative method was 100%. The semi-quantitative method developed in this study only requires modelling based on a large number of samples from MRL, and the semi-quantitative results are determined by the Raman characteristic peak intensity. Compared to existing CBZ semi-quantitative analysis methods of CBZ, such as PLS-DA [42], this semi-quantitative does not require complex classification models and classification parameters such as the variable importance of variables in projection fraction, so it is simpler and more tractable. In order to ensure the SERS rapid detection method and semi-quantitative analysis method satisfy the evaluation standards of rapid detection methods (released by the State Food and Drug Administration in 2017). POD curve and dPOD curve were constructed for different methods or different labs within a certain concentration range, and the consistency of the methods was determined based on whether the POD is the same. The results showed that at the MRL level of CBZ (5 mg/kg), the SERS qualitative detection method was consistent among different labs, and the results were the same as the reference method. Compared with ‘Technology specification for the evaluation of food rapid detection products’ [37] , this evaluation method can show the change of sensitivity with concentration, and the LOD is well defined. It can compare the consistency of each concentration interval within the detection concentration range and can be applied to the consistency analysis between methods, environments, and instruments. The obtained POD curve can display the results visually, which is more statistically significant.

The semi-quantitative analysis method based on SERS technology can perform rapid primary screening of samples according to the characteristic peak intensity of CBZ at 630 cm^−1^ in samples, which can improve the detection efficiency, and can be extended to other finite contaminants such as melamine in liquid milk and other pesticide residues in food. However, the training set of the semi-quantitative model requires a large amount of sample size (the number of samples >50), which leads to consume most of the time and energy before modeling. How to use less time to obtain the more sample information and reduce the preparation time is the bottleneck and future development direction of this research. Raman hyperspectral imaging technology is an advanced non-destructive testing technology that combines conventional imaging and spectroscopy to collect Raman spectral information of each pixel in space, so as to conduct qualitative, quantitative and localized analysis of samples [43]. Compared with SERS, the advantage of Raman hyperspectral imaging technology is that it can continuously collect a large number of spectral information through an automated sample platform, so as to obtain more sample information in less time. For example, Yang et al. applied Raman hyperspectral imaging technology to continuously collect spectral information of 100 pixels in 10 min [44]. However, it would have taken at least 50 min to collect the spectral information with SERS. Therefore, the semi-quantitative method developed in this study can be combined with the Raman hyperspectral imaging to shorten the sample information acquisition time and further improve the efficiency of sample screening. 

## 4. Materials and Methods

### 4.1. Samples, Reagents and Instruments 

Sample: Apple (commercially available); Reagents: Carbendazim solid standard, ethanol (AR), dichloromethane (AR), NaCl solution (1 mol/L), nano-gold solution (Shanghai Oceanhood opto-electronics tech Co., Ltd., Shanghai, China).

Instruments: Portable Raman spectrometer (SEED 3000, Shanghai Oceanhood opto-electronics tech Co., Ltd., China), precision electronic balance (Sartorius, Germany), vortex mixer (VORTEX-GENIE2, Scientific Industries, New York, NY, USA), Eppendorf 5810R centrifuge (20050647GZ, Eppendorf, Germany).

### 4.2. Methods

#### 4.2.1. Sample Preparation

Apple pulp was put into a 50 mL tube, and it was broken up with a homogenizer. 2 g of pulp was weighed and added with 20 μL of standard solutions of CBZ with different concentrations, so that the concentrations of CBZ in samples were 5 mg/kg, 2.5 mg/kg, 1 mg/kg, 0.5 mg/kg, 0.1 mg/kg, 0.05 mg/kg. The samples with 3 mL of ethanol (5%, *v*/*v*) were vortexed for 3 min and centrifuged at 4000 rpm for 2 min. 2 mL of sample supernatant was mixed with 2 mL of dichloromethane in a 5.0 mL tube. After layering, 1 mL of liquid in the lower layer was taken into a gas-phase injection vial, dried with a nitrogen blower, and reconstituted with 500 μL of ethanol (20%, *v*/*v*). The reconstituted liquid was vortexed for 1 min and used for SERS detection.

#### 4.2.2. SERS Detection

50 μL reconstituted solution with 200 μL of nano-gold solution and 50 μL of NaCl solution (1 mol/L) were added to the detection vial. The samples were quickly mixed with a pipette and then tested on the SEED 3000. To avoid the aggregation of gold nanoparticles, the SERS detection was completed within 1 min. The excitation wavelength of the Raman spectrometer was 785 nm, the wavelength range of data acquisition was 200–4000 cm^−1^, the integration time was 1 s, and the laser power was 200 mw. Spectral data were collected by Uspecral-PRO software. (Shanghai Oceanhood opto-electronics tech Co., Ltd., Shanghai, China).

#### 4.2.3. Data Processing

(1) Screening of Raman characteristic peaks of CBZ in apple

Based on the positions of the Raman characteristic peak of CBZ standard, the Raman shift peaks (the number varies from 2 to 6) specific to the blank samples were selected as the characteristic peaks of CBZ, and a library of Raman characteristic peak was established. After obtaining the Raman spectral of the sample, the search for the first order derivative peak and matching of the characteristic peak were performed. When the Raman characteristic peaks of the sample had all the characteristic peaks of CBZ, it was determined that the sample contains CBZ. The tolerance range of Raman shift is 3–10 cm^−1^ can be considered as the same characteristic peak.

(2) Establishment and screening of semi-quantitative models

The semi-quantitative model of the target at a specific concentration (such as MRL, etc.) is used to determine whether the concentration of target in the sample exceeds a specific concentration. To establish a semi-quantitative model at a specific concentration, it is first necessary to determine the applicable concentration of the Raman detection method and to confirm that the discriminant concentration is within the detection range of the current method. In practice, it is necessary to obtain Raman spectral data of spiked samples at specific concentrations to establish semi-quantitative models at different characteristic peaks. Then the optimal semi-quantitative model was determined according to the fitting of Gaussian distribution and the discrimination of different additive concentrations. These steps for establishing a semi-quantitative model at a specific concentration are as follows:

1. The establishment of semi-quantitative models. Raman spectral data of spiked samples (number of samples > 50) at specific concentrations were collected as a training set for a semi-quantitative model. A histogram of the Raman intensity of each Raman characteristic peak at a specific additive concentration was obtained and the distribution of the intensity was viewed. If the intensity of the characteristic peak does not obey the Gaussian distribution, Raman intensity of the characteristic peak is not only affected by the random error of detection, so it is not suitable for the semi-quantitative model and should be eliminated. For the characteristic peaks whose Raman intensity follows a Gaussian distribution, calculate the intensity mean and standard deviation, determine the confidence level, and draw the confidence interval (CI). The Raman intensity corresponding to the lower limit of the confidence interval (CI > 95%) is used as the semi-quantitative threshold.
2. Screening of semi-quantitative models. Raman data of spiked samples (number of samples > 20) at low concentration, half of specific concentration and specific concentration were collected as the test set for the semi-quantitative models of different characteristic peaks. The threshold value of the semi-quantitative model was used to determine whether the concentration in the sample exceeds a specific concentration, the POD was calculated, and the semi-quantitative model score under different characteristic peaks was computed. The higher the score of the model, the more accurate the semi-quantitative model will be. The model with the highest score and the Raman intensity conforming to the Gaussian distribution was selected as the optimal semi-quantitative model.

The formula for calculating the score is as follows: S is the score of the semi-quantitative model, POD1 is the probability of detection at low concentration, POD2 is the probability of detection at half of specific concentration, POD3 is the probability of detection at specific concentration.

(1) $$S=2^{\left({\mathrm{POD}}^{3}-{\mathrm{POD}}^{2}-{\mathrm{POD}}^{1}\right)}\times 50$$

3. Result determination of semi-quantitative models. When the qualitative determination result is that the sample contains target substance, if the characteristic peak intensity of the target substance exceeds the semi-quantitative threshold, it is determined that the concentration of the target substance is not lower than a specific concentration. On the contrary, the concentration is lower than a specific concentration.
(3) Evaluation of method based on POD model

The evaluation of Raman rapid detection method based on POD model specifically includes three steps: establishment of POD model, determination of blind sample results and calculation of evaluation result.

1. Establishment of POD model. According to the qualitative results of samples at blank, low concentration, half of a specific concentration, a specific concentration, etc. (the number of samples > 20), the number of test samples, positive samples and negative samples at each concentration were counted. The POD was calculated, and POD curve of the POD changing with the added concentration was drawn.

2. Determination of blind sample results [45]. According to the qualitative discrimination results of spiked blind samples (the number of samples >20) in different labs and reference methods, the number of test samples, positive samples and negative samples under each concentration was counted. At the same time, the number of samples exceeding a specific concentration is calculated from the semi-quantitative identification of the low concentration, half of the specific concentration and the spiked sample at the specific concentration.

3. Calculation of evaluation result. The POD of qualitative and semi-quantitative methods was drawn, and it was established for the method evaluation system of Raman detection. Sensitivity, specificity, and LOD were evaluated by the POD model, and the consistency with the reference method and the inter-laboratory consistency were evaluated by the difference of POD (dPOD).

The POD and its confidence interval are calculated as follows: x is the number of positive samples with positive results detected, N is the total number of samples, POD is the probability of detection, LCL is the lower limit of the 95% confidence interval, and UCL is the upper limit of the 95% confidence interval:

(1) when x = 0,

POD=0,

LCL=0,

UCL = 3.8415/(N + 3.8415)(2)

(2) When x=N,

 POD=1,

(3)  LCL=N/(N+3.8415),

UCL=1;

(3) When 0 < x<N,

(4) POD=x/N,

(5) $$\mathrm{LCL}=\frac{x+1.9207-1.9600\sqrt{x-\frac{x^{2}}{N}+0.9604}}{N+3.8415}$$

(6) $$\;\mathrm{UCL}=\frac{x+1.9207+1.9600\sqrt{x-\frac{x^{2}}{N}+0.9604}}{N+3.8415}$$

The dPOD and its confidence interval are calculated as follows: dPOD is the difference of POD, I is the method to be evaluated/laboratory, II is the reference method/laboratory:

(7) $$\mathrm{dPOD}={\mathrm{POD}}_{I}\;-\;{\mathrm{POD}}_{II},$$

(8) $$\mathrm{LCL}=\mathrm{dPOD}\;-\;\sqrt{{\left({\mathrm{POD}}_{I}-{\mathrm{LCL}}_{I}\right)}^{2}+{\left({\mathrm{POD}}_{II}-{\mathrm{UCL}}_{II}\right)}^{2}}$$

(9) $$\mathrm{UCL}=\mathrm{dPOD}+\sqrt{{\left({\mathrm{POD}}_{I}-{\mathrm{UCL}}_{I}\right)}^{2}+{\left({\mathrm{POD}}_{II}-{\mathrm{LCL}}_{II}\right)}^{2}};$$

## 5. Conclusions

This study carried out qualitative and semi-quantitative analysis of CBZ in apple and the method was evaluated by a binary output-based validation system. The LOD of the qualitative method was 0.5 mg/kg. When the additive concentration was 5 mg/kg, the POD of the semi-quantitative method was 100%. When the added concentration was greater than 5 mg/kg, the SERS method and the reference method are consistent and the detection results are not affected by the lab. The threshold-based semi-quantitative method proposed can quickly determine whether the finite contaminants in blind samples exceed the MRL. Meanwhile, the evaluation method based on binary output provided a reference for the evaluation system of Raman spectroscopy rapid detection technology, which is of great significance for food rapid detection technology and has some significance in other rapid detection fields. 

## 6. Patents

The work reported in this manuscript resulted in a patent, which has been granted under the patent number ZL201911005222.1

## Figures and Tables

**Table 1:** Assignments and Raman shifts (cm^−1^) for SERS spectra of CBZ.

Wavenumber (cm^−1^)	Vibrational Description	Wavenumber (cm^−1^)	Vibrational Description
630	ring stretching and C-C bending	1315	ring stretching
728	C-C bending and C-O-CH_3_ bending	1370	C—N stretch
1000	C-N bending and C-C bending and C-O-CH3 stretching	1460	N-H bending and C-H bending
1218	C-C stretch, C-C bending and N-H bending	1523	N-H bending and C-N stretch
1260	C-H bending and N-H bending		

**Table 2:** The POD of CBZ in apple at different concentrations.

Concentration (mg/kg)	x	N	POD	LCL	UCL
0.000	0	10	0.000	0.000	0.280
0.050	0	50	0.000	0.000	0.070
0.100	40	50	0.800	0.670	0.890
0.500	39	40	0.975	0.871	1.000
1.000	40	40	1.000	0.910	1.000
2.500	40	40	1.000	0.910	1.000
5.000	40	40	1.000	0.910	1.000

Note: x is the number of positive samples with positive results detected; N is the total number of samples; POD is the probability of detection; LCL is the lower limit of the 95% confidence interval; UCL is the upper limit of the 95% confidence interval.

**Table 3:** Evaluation of the consistency between the Raman qualitative method and the reference method for CBZ in apple.

Method	SERS					HPLC					Difference in POD (dPOD)
Concentration (mg/kg)	x	N	POD	LCL	UCL	x	N	POD	LCL	UCL	
0.00	0	10	0.000	0.000	0.280	0	10	0	0.000	0.280	0.000
0.01	0	50	0.000	0.000	0.070	0	50	0	0.000	0.070	0.000
0.10	40	50	0.800	0.670	0.890	50	50	1	0.930	1.000	0.200
0.50	39	40	0.975	0.870	1.000	40	40	1	0.910	1.000	0.025
2.50	40	40	1.000	0.910	1.000	40	40	1	0.910	1.000	0.000
5.00	40	40	1.000	0.910	1.00	40	40	1	0.910	1.000	0.000

Note: x is the number of positive samples with positive results detected; N is the total number of samples; POD is the probability of detection; LCL is the lower limit of the 95% confidence interval; UCL is the upper limit of the 95% confidence interval.

**Table 4:** The POD of CBZ in apple by Raman qualitative method among different labs.

Lab	Ⅰ					Ⅱ					Difference in POD (dPOD)
Concentration (mg/kg)	x	N	POD	LCL	UCL	x	N	POD	LCL	UCL	
0.00	0	10	0.000	0.000	0.280	0	10	0.000	0.000	0.280	0.000
0.05	0	50	0.000	0.000	0.070	0	50	0.000	0.000	0.070	0.000
0.10	40	50	0.800	0.670	0.890	48	50	0.960	0.865	0.989	0.160
0.50	39	40	0.975	0.870	1.000	40	40	1.000	0.910	1.000	0.025
2.50	40	40	1.000	0.910	1.000	40	40	1.000	0.910	1.000	0.000
5.00	40	40	1.000	0.910	1.000	40	40	1.000	0.910	1.000	0.000

Note: x is the number of positive samples with positive results detected; N is the total number of samples; POD is the probability of detection; LCL is the lower limit of the 95% confidence interval; UCL is the upper limit of the 95% confidence interval.

**Table 5:** Scores for semi-quantitative models.

Concentration (mg/kg)	0.5		2.5		5		Score (S)
Peaks (cm^−1^)	x	POD1	x	POD2	x	POD3	
630	0	0	2	0.050	40	1	97
728	0	0	11	0.275	40	1	83
1000	8	0.200	39	0.975	40	1	44
1218	0	0	29	0.725	40	1	60
1260	9	0.225	40	1	40	1	43
1315	0	0	0	0	40	1	100

Note: x is the number of positive samples with positive results detected; POD is the probability of detection.

**Table 6:** Detection situation between different labs by semi-quantitative method for CBZ in apple.

Lab	Ⅰ					Ⅱ					Difference in POD (dPOD)
Concentration (mg/kg)	x	N	POD	LCL	UCL	x	N	POD	LCL	UCL	
0.50	0	40	0.000	0	0.088	7	40	0.175	0.087	0.320	0.175
2.50	25	40	0.625	0.470	0.758	34	40	0.850	0.710	0.930	0.225
5.00	40	40	1.000	0.912	1.000	40	40	1.000	0.912	1.000	0.000

Note: x is the number of positive samples with positive results detected; N is the total number of samples; POD is the probability of detection; LCL is the lower limit of the 95% confidence interval; UCL is the upper limit of the 95% confidence interval.

## Data Availability

The dataset used and/or analyzed during the current study are available from the corresponding author on reasonable request.

## Sample Availability

Samples of the compounds are not available from the authors.
